# Fabrication and desired properties of conductive hydrogel dressings for wound healing

**DOI:** 10.1039/d2ra07195a

**Published:** 2023-03-14

**Authors:** Lei Nie, Qianqian Wei, Jingyu Li, Yaling Deng, Xiaorui He, Xinyue Gao, Xiao Ma, Shuang Liu, Yanfang Sun, Guohua Jiang, Oseweuba Valentine Okoro, Amin Shavandi, Shengli Jing

**Affiliations:** a College of Life Sciences, Xinyang Normal University Xinyang 464000 China nieleifu@yahoo.com nielei@xynu.edu.cn +86-13600621068; b Université libre de Bruxelles (ULB), École polytechnique de Bruxelles, 3BIO-BioMatter, Avenue F.D. Roosevelt 50 - CP 165/61 1050 Brussels Belgium amin.shavandi@ulb.be oseweubaokoro@gmail.com; c College of Intelligent Science and Control Engineering, Jinling Institute of Technology Nanjing 211169 P.R. China; d School of Resources and Environmental Engineering, Wuhan University of Technology Wuhan 430070 P. R. China; e College of Life Sciences and Medicine, Zhejiang Sci-Tech University Hangzhou 310018 China; f School of Materials Science and Engineering, Zhejiang Sci-Tech University Hangzhou 310018 China; g International Scientific and Technological Cooperation Base of Intelligent Biomaterials and Functional Fibers, Zhejiang Sci-Tech University Hangzhou 310018 China

## Abstract

Conductive hydrogels are platforms recognized as constituting promising materials for tissue engineering applications. This is because such conductive hydrogels are characterized by the inherent conductivity properties while retaining favorable biocompatibility and mechanical properties. These conductive hydrogels can be particularly useful in enhancing wound healing since their favorable conductivity can promote the transport of essential ions for wound healing *via* the imposition of a so-called transepithelial potential. Other valuable properties of these conductive hydrogels, such as wound monitoring, stimuli-response *etc.*, are also discussed in this study. Crucially, the properties of conductive hydrogels, such as 3D printability and monitoring properties, suggest the possibility of its use as an alternative wound dressing to traditional dressings such as bandages. This review, therefore, seeks to comprehensively explore the functionality of conductive hydrogels in wound healing, types of conductive hydrogels and their preparation strategies and crucial properties of hydrogels. This review will also assess the limitations of conductive hydrogels and future perspectives, with an emphasis on the development trend for conductive hydrogel uses in wound dressing fabrication for subsequent clinical applications.

## Abstract 2

Conductive hydrogels are recognized as promising materials for wound healing. Valuable properties of conductive hydrogels suggest the possibility of their use as an alternative wound dressing to traditional dressings such as bandages.

## Graphical abstract

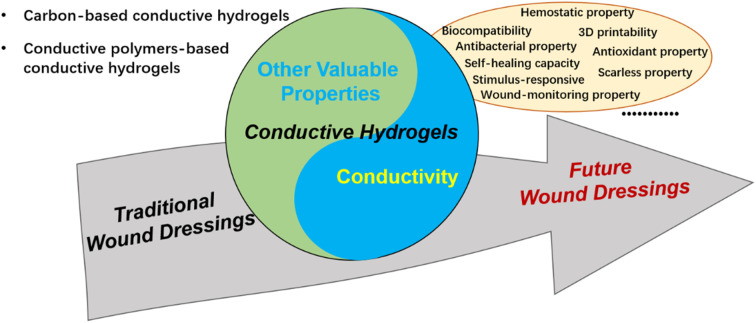

## 1 Introduction

**Fig. 1:**
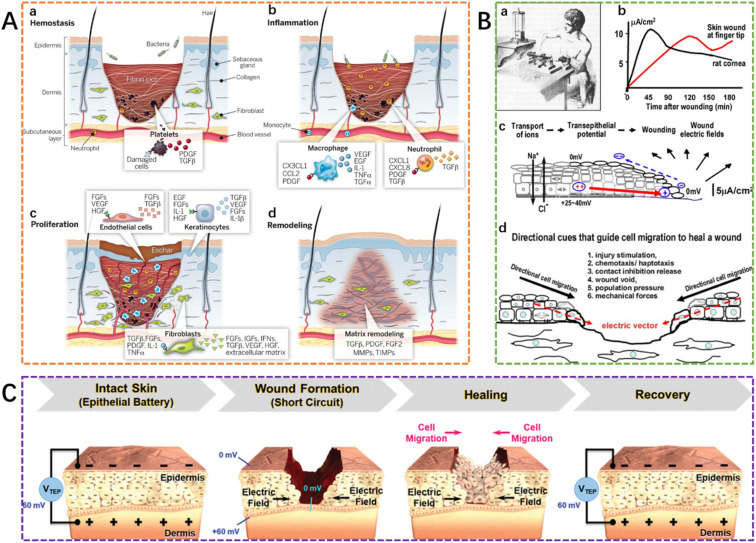
The processes of wound healing. (A) Wounding healing is divided into four stages: hemostasis, inflammation, proliferation, and remodeling.^9^ This figure has been adapted/reproduced from ref. 9 with permission from *Science*, copyright 2023. (B) The distribution of the electric current flow and the endogenous electric fields in a skin wound, and the directional cues could guide cell migration to heal a wound.^35^ These figures have been adapted/reproduced from ref. 35 with permission from *Seminars in Cell & Developmental Biology*, copyright 2023. (C) Trans-epithelial potential (TEP) and electric field at wound site before and after the healing process.^36^ This figure has been adapted/reproduced from ref. 36 with permission from *Advanced Healthcare Materials*, copyright 2023.

The skin is the largest organ of the human body and serves as a barrier that protects the body from external factors such as excessive evaporation of water, exposure to harmful chemical/radiological components and disease-causing pathogens, while simultaneously maintaining electrolyte balance and the retention of nutritional components within the body.^1–3^ The skin also is also sensitive to electrical signals due to its conductivity property that ranges from 2.6 mS cm^−1^ to 1 × 10^−4^ mS cm^−1^. Indeed, the conductive nature of the skin is reported to be crucial to the biological regulation within the human body.^4–8^ Due to the important role, the skin performs as a protective barrier, and it has the ability to repair itself to recover its structural and functional integrity after damage *via* wounds.^9^ The wound healing/repair process is a complex process composed of hemostasis, inflammation, proliferation, and remodeling stages involving diverse enzymes, growth factors and cytokines. These enzymes, growth factors and cytokines have important effects in modulating relevant cell activities to enable wound closure and tissue repair (Fig. 1A).^9^ However, such self-repair may not be efficient when large and severe wounds are involved, making it crucial to explore strategies to accelerate the wound healing process and also avoid scar formation.^4,10^ Hydrogels have therefore been proposed as useful tools that can aid wound closure and tissue repair. Hydrogel is a unique three-dimensional network structure composed of natural or synthetic polymers and is considered more beneficial to the wound healing process than traditional dressings such as bandages.^11,12^ This is because, these traditional dressings do not actively promote the healing process and have been reported to have the potential of adhering to the new forming skin tissue leading to the risk of second injury upon replacement.^4,13^ On the other hand, the hydrogel dressings actively promote wound healing by providing a moist environment, absorbing tissue exudates for a cleaner wound microenvironment and enhanced mass transfer of gas and nutrient molecules.^7,14–16^

Notably, it has been demonstrated that electroactive materials can affect the activities of electrically excitable cells such as fibroblasts and keratinocytes, which play a key role in the wound healing process.^7,17–23^ This implies that the application of materials with conductive properties could be employed to enhance cell adhesion, migration, proliferation and differentiation for the promotion of wound healing.^7,17–23^ Furthermore, under physiological conditions, there is a risk of the overproduction of reactive oxygen species (ROS) that can disrupt the cellular oxidant/antioxidant balance, enhancing the risk of infection, slow tissue regeneration and poor wound healing.^7,21^ The scavenging of the ROS using conductive materials can therefore facilitate the protection of tissues against oxidative damage during the regeneration processes by reducing the excessive levels of free radicals.^21^ Recognizing the potential of these conductive materials, some researchers have proposed the integration of conductive materials into the hydrogel dressings, to facilitate the production of so-called conductive hydrogel dressings. Indeed, preliminary experimental results have shown that these conductive hydrogel dressings not only accelerate wound closure and new skin tissue regeneration but also enable the treatment of wounds of different types and complexities.^2,7,8,24–31^ For instance, Stubbe *et al.*, demonstrated the potential of employing conductive hydrogels based on hydroxylated multiwall carbon nanotubes (CNT-OH), in the treatment of burn wounds.^32^ Recognizing the limitations of conventional wound dressings (*i.e.* based on polyester, rayon *etc.*) such as their inability to effectively ‘drain wound’ and accelerate epithelial cell migration for the promotion of granulation tissue development, the current review seeks to explore the conductive hydrogels, with a special emphasis on their wound healing applications. To this regard, this review focused on the categorizations of conductive hydrogels for wound dressing fabrication, their preparation methodologies, and the strategies reported in the literature for their utilization in different wound types. We also highlighted the unique functionality and advantages of conductive hydrogels-based wound dressings while also briefly discussing their limitations. This review finally concludes by presenting the future trends and opportunities for the development of multifunctional wound dressings using conductive hydrogels.

## 2 Functionality of conductive hydrogel dressings in wound healing process

In intact skin, there is sustained directional transport of ions *via* active Na^+^/K^+^ ATPase pumps, which actively pump Na^+^ inward to the basal side and Cl^−^ out to the apical side, of the epithelial tissues. The epidermis, therefore, generates a voltage, called the transepithelial potential (TEP) or “skin battery” or “epidermal battery”,^33–35^ which ranges from 10 mV to 60 mV, depending on the region measured, between the top of the stratum corneum (the outermost layer of the skin) and the bottom of the epidermis.^36^ However, when a wound occurs, ion leakage occurs across wounded cells or cell layers, leading to epithelial barrier disruption, and the subsequent epidermal battery is ‘short-circuited’.^35,37^ At a wound site through the epidermis, the TEP drops to zero, and in this process, the movement of electric charges leads to a naturally occurring endogenous electric field and electric current.^33^ The wound endogenous electric currents were measured over 150 years ago by Emil Du Bois-Reymond (1818–1896), a German physiologist.^33,35,36^

Fig. 1B also shows the distribution of the electric current flow and the endogenous electric fields in a skin wound. The wound-closed electric current flow is orientated towards the wound from the unwounded epidermis with the endogenous electric field mainly distributed near the wound edge, such that its intensity falls from 100 V m^−1^ at the wound edges to 0 V m^−1^ at the wound center.^33^ This field provides a directional cue which can direct cell migration for wound healing (Fig. 1C).^36^Fig. 1C depicts the dynamic change of the TEP and electric field at the wound site during the wound healing process. The endogenous electric fields therefore play a vital role in wound healing and regenerative processes.^38^ Many studies have shown that at the wound site, the stimulation of the endogenous current is effective for the promotion and acceleration of wound closure since cell migration of fibroblasts, keratinocytes, macrophages, and neutrophils, is enhanced. Although the application of exogenous electrical stimulation may also translate to similar favorable effects, the requirement of larger external electronics may require complex and time-consuming operations, which can be inconvenient to patients and doctors.^33,39^ Interestingly, it has been demonstrated that conductive dressings can effectively stimulate endogenous current^2,7,8,24–30^ since they can influence the wound TEP by forming a wound-closed electric current flow while also promoting cell migration for enhanced wound healing.^7,31^ These conductive dressings can promote the transfer of the electro-signals to the wound site and thus aid the activation of the cellular activity with the electroactive compounds in the conductive hydrogel dressings.^7,31^ In addition to the unique properties of conductive hydrogels that promote endogenous current flow, since they are hydrogels, these dressings have the capacity to promote the rehydration of wound beds and facilitate autolytic debridement while also limiting necrosis.^40–42^ Inherent antioxidant properties of conductive polymers in the conductive hydrogel dressings can also promote wound healing.^31^

## 3 Types of conductive hydrogels used in wound dressings

Conductive hydrogels may be classified based on the matrix materials properties of the hydrogels, and the conductive materials that are responsible for the basic hydrogel properties and their conductivity properties, respectively. To this regard, this section will explore major types of conductive materials with their fabrication strategies discussed.

### 3.1 Carbon-based conductive hydrogel dressings

#### 3.1.1 Carbon nanotube

**Fig. 2:**
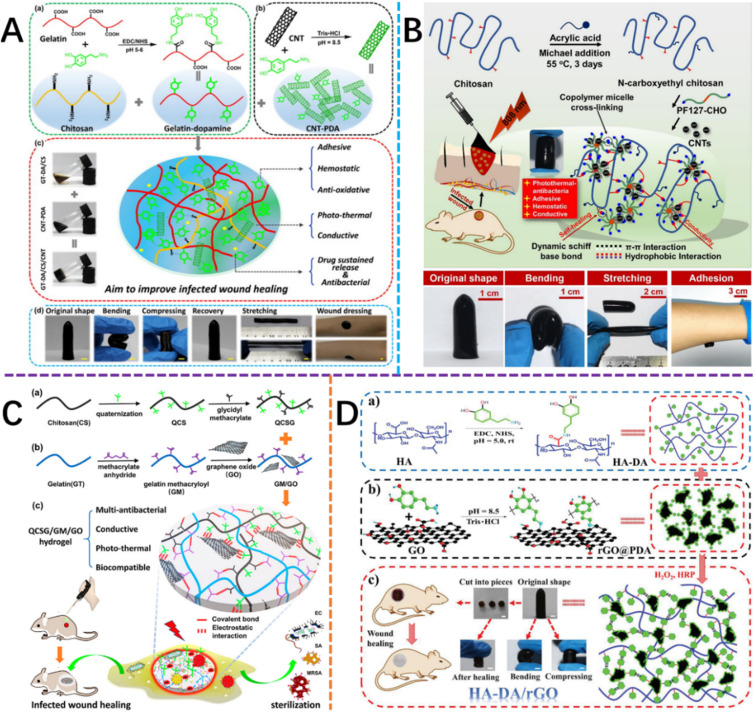
The conductive materials could be incorporated into hydrogels as wound dressings. (A) CNT.^28^ This figure has been adapted/reproduced from ref. 28 with permission from *Journal of Colloid and Interface Science*, copyright 2023. (B) GO.^27^ This figure has been adapted/reproduced from ref. 28 with permission from *Chemical Engineering Journal*, copyright 2023. (C and D) rGO.^26,29^ These two figures have been adapted/reproduced from ref. 26 and 29 with permission from *Biomacromolecules* and *Small*, respectively, copyright 2023.

Carbon nanotubes (CNTs) are allotropes of carbon that are characterized by a nanostructure with a length-to-diameter ratio of >1 000 000.^43^ They are also characterized by large surface areas, and excellent mechanical, stable photothermal and electronic properties.^27^ When CNTs are added to the hydrogel, the mechanical properties of the hydrogels will be enhanced. Additionally, the introduction of CNTs will also enhance the biological activity of the hydrogel when employed as a wound dressing *via* the incorporation of favorable interactions between CNTs and cells, biomolecules and native tissue, while also increasing cell migration in hydrogels.^44^ Electrical signals around the wound may also be mediated by the introduction of CNTs for the promotion and acceleration of wound repair and tissue regeneration by further promoting cell migration and blood flow.^28^ The incorporation of CNTs in wound dressings was demonstrated in the study by Liang *et al.*^28^ In the study, a layer of polydopamine (PDA) was initially used to coat the surface of the CNT and reduce the strong hydrophobic interactions that characterize CNTs (Fig. 2A). The modified CNTs were then added to hydrogels such that a positive correlation between increasing CNT concentration and hydrogel conductive properties was determined. Indeed, the study was able to show that the conductivities of hydrogels improved from 2.5 × 10^−2^ S m^−1^ to 7.2 × 10^−2^ S m^−1^ (*i.e.* human skin conductivity) as the concentration of the CNT in the hydrogel increased from 0 to 4 wt%.

The study also showed that when the CNT-laden hydrogel was applied to full-thickness wounds on the skin of a mouse model, favorable antibacterial, antioxidant and adhesive effects were observed. Similar studies involving the incorporation of CNTs in hydrogels for improved biological properties were undertaken by Liang *et al.*,^28^ and He *et al.*,^27^ with favorable antibacterial behaviors both *in vitro* and *in vivo* against Gram-(+) *Staphylococcus aureus* (*S. aureus*) and Gram-(−) *Escherichia coli* (*E. coli*) micro-organisms. The improved biological properties observed when CNTs were introduced in hydrogels further highlight the potential of its utilization in the treatment of infected wounds.

#### 3.1.2 Graphene

Graphene is a two-dimensional conductive material characterized with favorable biocompatibility, mechanical strength, high surface area, and thermal and electrical conductivity properties that enhance its potential utilization for biomedical applications.^45^ Graphene is also characterized by a high specific surface area (*i.e.* 2630 m^2^ g^−1^ for monolayer) which, when introduced to hydrogel, may enhance the bioactivity of hydrogel *via* improved interactions between the biomolecules, cells, and human tissues as demonstrated in the literature.^46,47^ For instance,^48^ showed that the composite hydrogel composed of graphene/Ag (mass ratio of 1 : 5) and based on the cross-linking reaction of graphene with acrylic acid and methylene bisacrylamide, enhanced healing rates when applied on wounds present on rat models such that a 15 day period was sufficient to facilitate complete tissue reconstruction. The ability of graphene to enhance the antibacterial activity of hydrogels was demonstrated in the study by ref. 49. In the study, bacterial cellulose (BC) was loaded with ∼11.7 wt% of graphene quantum dots with the resulting hydrogel composite shown to be not only biocompatible but also inhibited the growth and proliferation of *Staphylococcus aureus* and *Streptococcus agalactiae* and also showed antibacterial effects against Methicillin-resistant *Staphylococcus aureus*, *Escherichia coli*, and *Pseudomonas aeruginosa*. This graphene-based hydrogel was also shown to promote wound healing *via* enhancing wound fluid absorption and water retention and promoting the migration of human fibroblasts, thus promoting angiogenesis. Another study employed graphene in the production of near-infrared (NIR)-responsive hydrogels, with the graphene-based hydrogel shown to be able to efficiently capture and release cells, in response to NIR stimulus.^50^ In spite of the advantages of graphene-based hydrogels, their application in the biomedical field may be limited due to the poor mechanical properties of the composite hydrogels, which arises as a result of the physical interactions between graphene sheets and the difficulty in controlling temperature and critical pH value.^51^ Further work is therefore required in the area.

#### 3.1.3 Graphene oxide

Graphene oxide (GO) is an excellent conductive material and a monoatomic layer with a large surface area that is capable of aiding the condensation of cationic polymers by electrostatic interaction due to the high electronegativity in the surface.^52^ Thus cationic polymers such as glycidyl methacrylate functionalized quaternized chitosan can be condensed on the GO surface for enhanced bioactivity of graphene-based hydrogels *via* the unique interaction patterns between GO and cells, biomolecules and even tissues in a body.^53^ Additionally, existing hydrophilic groups on the GO surface, such as epoxy group, hydroxyl and carboxyl groups, can be chemically modified for better water dispersion than graphene.^54^

The antibacterial activity of GO has also been reported, with this activity hypothesized to be due to the induction of oxidative stress on bacterial membranes due to the sharp edges GO.^55^ In recognition of these unique advantages of GO, several studies have explored the potential of employing GO in the fabrication of conductive hydrogels.^26,56^ For instance, in the study undertaken by ref. 56, a GO-based conducting composite hydrogel was prepared. The preparation of the GO-based conducting hydrogel was achieved *via in situ* chemical polymerization of aromatic monomers such as pyrrole, 3,4-ethylenedioxythiophene and aniline in GO sheets. The study was able to demonstrate that the GO-based hydrogel containing polymers of pyrrole presented favorable storage, high storage moduli of >10 kPa, and strong electrochemical activity.

Further investigations by Liang *et al.*,^26^ showed that GO-based conductive hydrogels could be used in the repair of infectious skin tissue defects. In the study, drug-resistant Methicillin-resistant *Staphylococcus aureus* (MRSA) infected mouse full-thickness defect model was treated with the GO-based conductive hydrogels, with the conductive hydrogel shown to exhibit better-wound healing effect than the hydrogel without conductive components. The electrical and photothermal properties of GO played an important role in accelerating wound healing (Fig. 2B).

#### 3.1.4 Reduced graphene oxide

Reduced GO (rGO) has good water dispersion and better conductivity than GO and has also been reported to enhance cell adhesion and proliferation.^29,57^ The ability of rGO to promote cell adhesion and proliferation was demonstrated by ref. 57. In study,^57^ GO-based conductive hydrogels were prepared *via* the reduction of GO and subsequent ‘bridging’ using l-cysteine. Wang *et al.* showed, *via in vitro* experiments, that the rGO-based hydrogel that was bridged by l-cysteine molecules promoted excellent cell adhesion and growth, thus suggesting the future functionality of employing these hydrogels as conduits for the provision of electrical stimulation for enhanced cell adhesion and growth. Crucially, rGO could also serve as a new type of antibacterial agent,^29^ thus providing excellent anti-bacterial properties when incorporated into hydrogels as wound dressings.^58^ For instance, in the study by ref. 58, a hybrid polyvinyl alcohol (PVA) hydrogel that contained rGO/MoS_2_/Ag_3_PO_4_ composites, was developed, *via* a two-step process involving hydrazine reduction and *in situ* deposition, followed by hydrogel preparation using the freeze–thaw cycle method. The study was able to show that the hybrid hydrogel containing rGO exhibited antibacterial properties against *Staphylococcus aureus* (*S. aureus*) and *Escherichia coli* (*E. coli*) while retaining favorable biocompatibility properties. Indeed, no toxic effects on cell viability and cell morphology, were observed. This study highlighted that the rGO based conductive hydrogels presented potential in promoting antibacterial wound healing. Similarly, Liang *et al.*,^29^ investigated the preparation of conductive photothermal antibacterial hydrogels that contained rGO and hyaluronic acid-*graft*-dopamine. The study showed that the rGO-based conductive hydrogels were beneficial for full-thickness skin repair and regeneration during wound healing (Fig. 2C).

Zhang *et al.*,^25^ prepared a kind of supramolecular hydrogels to enhance complete skin regeneration by adding rGO, quaternized chitosan-*graft*-cyclodextrin, and quaternized chitosan-*graft*-adamantane into the hydrogel. Li *et al.*,^2^ relying on polydopamine-coated reduction graphene oxide (rGO–PDA), quaternized chitosan and poly(*N*-isopropylacrylamide) (PNIPAm), prepared a series of multifunctional wound dressings to accelerate wound closure and wound healing.

### 3.2 Conducting polymers-based conductive hydrogel dressings

Conducting polymers (CPs) such as polypyrrole (PPy), polyaniline (PANI), aniline oligomers, and polythiophene are characterized by favorable electrical properties that have been shown to aid the wound healing process by promoting cell proliferation, migration, adhesion, and differentiation.^4^ However, it is challenging to fabricate conductive wound dressing hydrogel using pure CPs due to its poor mechanical and solubility properties.^8,35^ In response to these limitations, researchers have therefore sought to investigate the tailoring of CPs to enhance their mechanical properties, biocompatibility, electrical conductivity and solubility *via* the application of chemical and/or physical modifications.^35^ These modifications subsequently facilitated their use in the preparation of conductive hydrogels, as discussed in the subsequent sections.

#### 3.2.1 PPy-based conductive hydrogel dressings

As a bioorganic conducting polymeric, polypyrrole (PPy) is regarded as the most studied conductive polymer since it possesses electrically conductive features, favorable stability and excellent absorbance properties around the near-infrared (NIR) region, which makes it particularly useful in the preparation of conductive hydrogel dressings.^59^ PPy is typically synthesized from pyrrole *via* chemical oxidation or by electrochemical oxidation using a radical initiator and an electrolyte solution or an electrolyte solution with the platinum-coated electrode, respectively.^60^ This PPy can be employed in the preparation of hydrogels to enhance their conductivity, self-healing and mechanical properties, as demonstrated in the study by ref. 61. In the study, a hydrogel was initially made from polyurethane (PU) while employing 4-aminophenyl disulfide (APDS) as the chain extender, after which PPy was incorporated *via in situ* polymerization. The introduction of PPy facilitated the optimal conductivity property of the hydrogel of 0.055 S m^−1^, without compromising the self-healing property, mechanical properties of tensile stress (1.1 MPa), flexible stretchability (>500%), and mechanical recovery (10 min). Similarly, Yang *et al.*,^62^ showed that a PPy-based conductive hydrogel devised from hyaluronic acid was characterized by favorable conductive properties of 7.3 mS cm^−1^. The mechanical property was also enhanced with a 5-fold increase in Young's modulus reported compared to PPy-free hyaluronic acid hydrogels. It has also been reported that PPy can support cell adhesion, proliferation and differentiation when used in the preparation of conductive hydrogels.^6^ For instance, it was reported that conductive hydrogel preparation *via in situ* encapsulation of PPy nanorods inside the chitosan molecular framework was also shown to enhance biocompatibility and wound healing.^63^

#### 3.2.2 PANI-based conductive hydrogel dressings

**Fig. 3:**
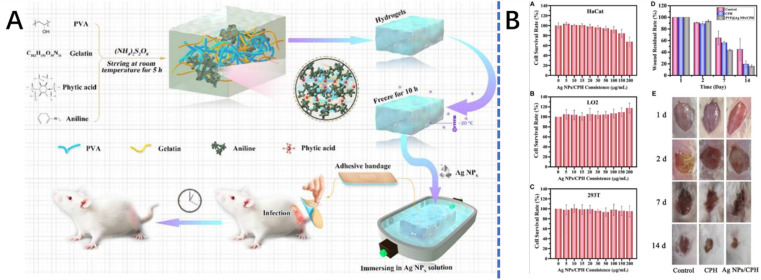
The synthesis process and applications of the Ag NPs/conductive polymer-based hydrogel.^66^ This figure has been adapted/reproduced from ref. 66 with permission from *Frontiers in Chemistry*, copyright 2023.

Polyaniline (PANI) is a polymer that contains a phenylene ring that has a –NH– group on either side.^64^ It is prepared by the oxidative polymerization of aniline *via* a 1,4-coupling of the monomer and under the action of a protonic acid.^64^ The resulting protonation is responsible for the transition from insulator-to-conductor transition, without compromising the number of π-electrons in the chain.^64^ As a conductive polymer, PANI is characterized by favorable biocompatibility and conductivity properties which are a function of its hydrophilic nature, low toxicity, good environmental stability, and nanostructured morphology. It, however, possesses unfavorable properties of low processability, infusibility, poor solubility^4,7^ and low degradability. These unfavorable properties may be reduced by blending with biopolymers and nanomaterials as demonstrated in the literature.^65^ For instance, Zhao *et al.*,^7^ prepared a PANI-based conductive hydrogel *via* the grafting of polyaniline onto quaternized chitosan (biopolymer) backbone with the resulting hydrogel employed as a wound dressing. The conductive hydrogel had good water solubility, good electroactivity, enhanced antibacterial activity and cytocompatibility and good free radical scavenging capacity. Xiao *et al.*,^66^ prepared a PANI-based conductive hydrogel for use in wound dressing for severely infected wounds by incorporating silver nanoparticle-doped conductive PANI into polyvinyl alcohol and gelatin hydrogel with excellent antibacterial properties (Fig. 3).

#### 3.2.3 Aniline oligomer-based conductive hydrogel dressings

Aniline oligomers or oligoanilines may be categorized into three main groups namely; Parent oligoanilines, which refer to compounds with ending groups of Ph/NH_2_, amino-capped oligoanilines which refer to compounds with ending groups of NH_2_/NH_2_; and phenyl-capped oligoanilines which refer to compounds with ending groups of Ph/Ph.^67^ These aniline oligomers are characterized by good electroactivity with the presence of a quinone ring of aniline oligomers capable of scavenging the free radicals, thus avoiding the disruption of high concentration reactive oxygen species (ROS) when introduced at the wound site.^8^ For instance, Haitao Cui *et al.*,^68^ showed that adding aniline tetramer to the antioxidant copolymer can prevent cell damage and increase cell viability *via* scavenging the excess ROS *in vitro*. Reza Gharibi *et al.*, also demonstrated that wound dressings containing antioxidant aniline tetramer moieties were endowed with a broad range of antimicrobial activities against different microorganisms and can promote *in vivo* wound healing by stimulating cell growth and proliferation of fibroblast.^69^ Qu *et al.*,^8^ also prepared a wound dressing conductive hydrogel with multifunctional including degradable, antibacterial, antioxidant and injectable properties *via* synthesizing oxidized hyaluronic acid graft phenyl/amino-capped aniline tetramer/*N*-carboxyethyl chitosan hydrogel.

#### 3.2.4 PEDOT-based conductive hydrogel dressings

PEDOT possesses a better higher conductivity and thermal stability than PPy.^4^ It has been chosen to fabricate many types of conductive films other than conductive hydrogel applied to wound healing. Li *et al.*,^70^ using the conductive material poly(3,4-ethylenedioxythiophene):poly(styrene sulfonate) and guar slime, prepared a kind of conductive wound dressing hydrogel with the injectable and rapid fabrication of self-healing to promote wound healing at the site of the movement like elbows, nape, wrists and knees, which easily undergo delayed healing and poor healing because of the interference of their frequent motion in daily life.

#### 3.2.5 Other conductive hydrogel dressings

Other conductive hydrogel dressings can be based on nanometer-sized metal and metal-oxide particles that are ultrafine and are widely known for their favorable conductivity, magnetic, catalytic and optical properties. These metal and metal oxide nanomaterials include noble metals like gold or silver and iron oxide or zirconia oxide, respectively.^60,71^

Conductive hydrogels based on gold nanoparticles incorporate the favorable properties of both hydrogels (*i.e.* matrix for cell proliferation) and gold (*i.e.* conductivity property). For instance, in the study undertaken by ref. 72, a conductive hydrogel composed of hyaluronic acid/gelatin/gold was prepared to promote surgical translation and bioprinting. This hydrogel was prepared *via* the combination of citrate–gold nanorods (GNRs) characterized with high aspect ratio, with hyaluronic acid and gelatin hydrogel. In the study, an indirect ligand exchange was employed between the cytotoxic surfactant for nontoxic citrate to fabricate nontoxic citrate–GNRs.^72^ The study was able to demonstrate that the gold-based conductive hydrogel could support the proliferation of seeded rat neural stem cells *in vitro*, *via* promoting cell adhesion and viability and thus provided a platform for the synergistic integration of gold for the conductive hydrogel preparation and use as wound dressing for improved surgical recovery.^72^

Similarly, in another study by ref. 73, a conductive hydrogel based on gold nanoparticles was investigated for its wound healing potential. In the study, gold nanoparticles were synthesized and used in the preparation of conductive hydrogels of polyethene glycol (PEG)–gold nanorods and cationic poly allyl amine hydrochloride–gold nanorods. These conductive hydrogels were observed to show enhanced wound healing properties, such as improved skin re-epithelization and antibacterial properties against *Staphylococcus aureus* and *Pseudomonas aeruginosa*. These gold-based conductive hydrogels, therefore, presented a promising platform for future skin wound healing applications.^73^ The noble metal of silver has also been employed in the preparation of conductive hydrogels since it can be used to enhance conductivity and antibacterial properties.^60^ For instance, hydrogels containing dispersed silver nanoparticles, synthesized using *Mimosa tenuiflora* extract as a reducing agent, were prepared in the study by ref. 74. The study showed that, the silver-based conductive hydrogel could promote favorable bactericidal effects against *Staphylococcus aureus* and *Escherichia coli* and favorable anti-inflammatory effects when applied in wound dressings for second-degree burn injury on Wistar rats, thus highlighting its potential to enhance effective recovery when used in wound dressings. Metal oxides such as iron oxide have also been employed in the fabrication of conductive hydrogels for wound dressing applications, as shown in the study. In the study,^75^ prepared a novel multi-component metal oxide-based conductive hydrogel by combining iron-oxide nanoparticles, that were impregnated in bacterial cellulose with alginate/casein hydrogels. The study showed that the incorporation of iron oxide into the hydrogel enhanced mechanical properties, and increased porosity and magnetic properties for promoting sustained drug delivery at wound sites. The multi-component hydrogel hybrid alginate–casein hydrogels containing iron oxide nanoparticles was proposed to constitute a facile approach that had potential wound healing applications.^75^

Having highlighted the major types of conductive hydrogels, some useful crosslinking systems and their applications are presented in Table 1.

**Table 1:** Summary of some major applications of CHs and their associated hydrogel-crosslinking systems^a^

Categorizations of CHs	Examples of CMs	Some studies on some possible hydrogel-crosslinking systems	Applications	References
Carbon-based CHs	Graphene/graphene oxide (GO)/rGO	In a study, sodium tetraborate (borax) was employed as the crosslinking method, with poly(vinyl alcohol) and dopamine hydrochloride employed in providing the hydrogel matrix laden with GO	This conductive hydrogel can be used in the production of soft strain sensors to monitor human movement	76
		Another study also reports that a conductive hydrogel based on the polyvinyl alcohol matrix laden with GO was prepared using both physically and chemically cross-linked networks using the synergy of freezing and salting (NaCl) out. The physical crosslinking was based on the PVA chains entangling with the GO during the salting-out process. The chemical crosslinking was due to electrostatic attraction caused by ionic charges of Na^+^ and Cl^−^ distributed inside the hydrogel	Strain sensors can be manufactured using this conductive hydrogel	77
		A graphene-based hydrogel was also prepared in the literature *via* polymerization *in situ*. The hydrogel matrix was prepared from *N*-isopropylacrylamide monomers with *N*′,*N*′-methylene-bisacrylamide as the crosslinker. *In situ* polymerization of super elastic graphene aerogel was then achieved using potassium persulfate as initiator and *N*′,*N*′-tetramethylenediamine as catalyst	Stimuli-responsive hydrogels can be used as sensors in biomedical devices	78
	Carbon nanotubes (CNTs)	Microgel particles were employed as dispersant and macro-crosslinker for producing covalently interlinked doubly crosslinked microgel/CNT composites *via* the free-radical reaction	Can be used for advanced soft-tissue repair	79
Conductive-polymer based CHs	Polyaniline (PANI)	Phytic acid facilitates crosslinking by acting as the gelator and dopant for the direct formation of a conducting polymer network. For instance, phytic acid facilitates crosslinking of PANI by protonating the nitrogen groups on PANI, leading to the formation of a mesh-like hydrogel network	Can be used for producing energy storage electrodes	80
		Chemical polymerization using chemicals (*i.e.* poly(ethylene glycol) diglycidyl ether, poly(styrene sulfonate) *etc.*) to crosslink PANI. In this approach, supramolecular self-assembly is achieved between positively-charged PANI chains and negatively-charged polyelectrolyte chains to induce gelation	Can be used for producing supercapacitors	81
	PEDOT	Ionic crosslinking using multivalent metal ions (Fe^3+^ or Mg^2+^). In other words, gelation is induced *via* electrostatic charges	Can be used in the production of sensors and electrodes	82 and 83
	Polypyrrole (PPy)	Synthesis *via* an interfacial polymerization method while using phytic acid as the crosslinking agent	Can be used in the production of electrodes	84
		*In situ* oxidative polymerization of pyrrole (Py) monomers using phytic acid and ammonium persulfate as dopant and crosslinker, respectively	Strain sensor to monitor human movement	85
Metal/metal oxide based CHs	Fe^3+^ (from (FeCl_3_·6H_2_O)	In this study, the cross-linking and polymerization of Fe^3+^ were achieved using acrylic acid (AA) and acrylamide (AM) to produce the conductive hydrogel designated as P(AA-*co*-AM)/Fe^3+^	Electronic skin	86
			Wearable electronics	
	Gold	Thermal crosslinking was employed in the preparation of gold-based conductive hydrogel using collagen as the hydrogel matrix	Can be employed to enhance tissue regeneration	87
		Ultraviolet crosslinking was employed in the preparation of gold-based conductive hydrogel using GelMA as the hydrogel matrix	Can be used in biomedical applications for the promotion of cardiac differentiation of embryoid bodies	88
	Silver	It was reported that a silver-based conductive hydrogel could be prepared by dispersing silver nanoparticles in polyacrylic acid–methylol urea (MU). In the study, crosslinking was achieved *via* noncovalent interactions between polyacrylic acid and MU, with the resulting crosslinked cage-like structures produced used in facilitating the stabilization of the silver nanoparticles	This conductive hydrogel has several applications such as in the manufacture of nanoelectronic devices and the fabrications artificial muscles	89

a CH and CM denote conductive hydrogel and conductive materials, respectively.

While Table 1 highlights several applications of conductive hydrogels, it must be emphasized that their use in wound healing (*i.e.* as wound dressing, wound monitoring *etc.*) constitutes the focus of this review. Thus, other uses (*i.e.* as electrodes) have been highlighted (Table 1) to demonstrate the extensive applicability of conductive hydrogels and will not be discussed further in this paper. Additionally, although conductive hydrogels have a wide range of functionalities, their limitations must also be acknowledged. For instance, conductive hydrogels have potential toxicity when employed *in vivo*, present a risk of polymerization residue and may also be nonbiodegradable.^90^ To address these limitations, several studies have explored the possibility of designing new conductive hydrogels that combine good biodegradability and good conductivity properties. For instance, the utilization of chemical modification of monomers as well as doping using precise counter ions, or bio dopants, that are capable of enhancing biocompatibility and biodegradability of the conductive hydrogels was proposed in the literature.^91^ The literature proposes that it is possible to enhance the biodegradability of conductive hydrogels *via* copolymerization of modified electroactive oligomers of conducting polymers using degradable ester linkages. Alternatively, conducting polymers such as PANI could be combined with biodegradable polymers such as poly (lactic acid) (PLA) and use ester linkages to enhance overall biodegradability.^92^ These materials constitute a new generation of biocompatible and biodegradable conductive hydrogels with their difficulty in design and development recognized. It is also hypothesized that it may also be possible to optimize the conductive property of the conductive hydrogel while avoiding potential biocompatibility issues *via* executing proper experimental designs. Clearly, more studies are required in the area to ensure that modifications to enhance biocompatibility and biodegradability do not hinder the future applicability of conductive hydrogels.

#### 3.2.6 Methods employed in the preparation of conductive hydrogels

These conductive hydrogels are commonly prepared *via* UV crosslinking with the *in situ* polymerization of hydrogel monomer achieved *via* the reduction of metal ions, using a reducing agent.^93^ The preparation methods facilitate the integration of metal centers to the polymer chain or as groups at the polymer side chains, while the linkages responsible for coupling are either reversible and dynamically coordinated or strongly covalent.^94^ The development of such metal-based conductive hydrogels was demonstrated in the study.^95^ In this study, a liquid metal nanoparticle and a cross-linked poly(acrylic acid) backbone with poly(3,4-ethylenedioxythiophene):sulfonated bacterial cellulose nanofiber nanomaterials were employed in the preparation of a conductive hydrogel. This conductive metal-based hydrogel was shown to have antibacterial properties against *Escherichia coli* and *Staphylococcus aureus* and favorable stretchability of 2850%. Other conductive hydrogels have also been prepared based on nanomaterials such as MXene nanosheets^96^ and ion-conducting hydrogels.^97^ Furthermore, the conductive hydrogels discussed above could also be classified as hybrid, acid/salts, electron and ion-conductive hydrogels, which refer to hydrogels composed of several conductive components, acids/salts and ions for electron transfer, respectively.^98–100^

The methods that can be used in the preparation of the conductive hydrogels discussed above can be categorized into three main methods which are based on the source and nature of the conductivity of hydrogel. These methods include *in situ* polymerization, post-polymerization and composite strategies.^101^ For instance, in the study by Zhao *et al.*,^102^ the *in situ* polymerization strategy was employed in the preparation of a conductive hydrogel. In the study, grafted monomers of aniline to quaternized chitosan were polymerized *via* using oxidized dextran as a dynamic Schiff crosslinker leading to the production of a conductive hydrogel. The conductive hydrogel exhibited antibacterial properties against *Escherichia coli* and *Staphylococcus aureus in vitro*, with a kill rate of 95% and 90% for *E. coli* and *S. aureus*, respectively. The conductive hydrogel was shown to have a conductivity of 0.43 mS cm^−1^ while also enhancing the proliferation of myoblasts. The post-polymerization strategy was demonstrated in the study by Wu *et al.*^103^ In the study, the hydrogels made from gelatin methacrylate were initially immersed in ammonium persulfate (APS) solution, after which the APS-laden hydrogels were incubated in the aniline solution, such that the APS solution functioned as an oxidant for aniline polymerization. The process facilitated the production of conductive polyaniline (PANI)–GelMA hydrogels. The conductive hydrogel was determined to have favorable cell adhesion properties and electrical properties (*i.e.* impedance of 2.9 ± 0.3 kΩ). The composite strategy was demonstrated in the study by Maharjan *et al.*,^104^ in which a conductive hydrogel was prepared by incorporating gold/silica hybrid (Au/SiO_2_) nanoparticles into a GelMA hydrogel. The study showed that the introduction of gold nanoparticles to the acellular matrix enhanced myocardial cell adhesion, proliferation and differentiation as well as conductivity. Some discussions regarding the highlighted strategies of *in situ* polymerization, post-polymerization and composite strategies are summarized in Table 2.

**Table 2:** Different strategies involved in the fabrication of conductive hydrogels^101,105–107^

Fabrication strategy	Some notes
*In situ* polymerization	This is a one-step strategy that involves the direct introduction of the monomer of the conductive polymer to the matrix, followed by the introduction of the oxidant to enable polymerization while hydrogel gelation occurs. This approach typically enables polymerization to occur homogeneously, although concerns related to incomplete reactions, leading to cytotoxic effects, have been reported. This approach, therefore, requires a preliminary design of the chemical synthesis process to limit the risk of incomplete reactions
Post polymerization	In this strategy, the preformed hydrogel matrix is immersed in a solution of the monomer of the conductive polymer, followed by the formation of the conductive hydrogel *via* the introduction of the monomer-filled hydrogel to an oxidant solution. Similar to the *in situ* polymerization method, there is a potential for the retention of unreacted components (*i.e.* monomers), leading to cytotoxic effects. The strategy also requires an extra polymerization step
Composite strategies	This strategy involves the direct addition of conductive materials (*i.e.* metals, carbon, conductive polymers *etc.*) to the hydrogel. The dispersal of the conductive materials in the gel endows the hydrogel with conductive properties. This strategy has the benefit of facilitating the control of conductivity while avoiding the risk of cytotoxic effects arising from incomplete reactions. There is, however, a challenge to the heterogeneous distribution of conductive materials

## 4 Favorable properties of conductive hydrogel dressings

Conductive wound dressings should be capable of promoting wound healing, wound closure and reducing scar formation^7^ with their useful properties being favorable biocompatibility, and physical properties (*i.e.* mechanical properties, porosity, *etc.*). Conductive hydrogels should also possess favorable properties such as antibacterial capacity, hemostatic, adhesiveness and non-adherent, antioxidant property, self-healing capacity, injectability, stimulus-responsive, degradable, and reducing scar *etc.*^4,9,108,109^ Major useful properties of conductive hydrogel dressings are discussed in the sub-sections below.

### 4.1 Biocompatibility property

Favorable biocompatibility and non-toxicity property are required properties of the conductive hydrogel dressing due to its direct contact with living cells and tissues during the wound healing process.^26^ The biocompatibility of the conductive hydrogel is typically measured *via* undertaking *in vitro* cytocompatibility investigations such as a hemolysis test or a direct contact test between hydrogels and the mouse fibroblasts (L929) cells.^26^ For instance,^26^ evaluated the biocompatibility of the conductive hydrogel dressings that were prepared as a composite of glycidyl methacrylate functionalized quaternized chitosan (QCSG), gelatin methacrylate (GM), and graphene oxide (GO). Their study was able to show that the conductive hydrogel had good biocompatibility and favorable effects on cell viability and histocompatibility. It is acknowledged that depending on the chosen conductive polymer and the approach employed in the synthesis of the hydrogel, conductive hydrogels may contain some toxic surfactants, implying that the synthesis approach must be optimized for enhanced biocompatibility, as demonstrated in ref. 110. In the study,^110^ the biocompatibility of PEDOT was enhanced *via* undertaking electrochemical polymerization around neurons.

### 4.2 Physical properties

Although conductive polymers have the potential to be modulated to achieve favorable morphological properties, their inherent mechanical rigidity limits their applicability as wound dressings.^111^ This is because a robust and elastic hydrogel wound dressing must have a favorable lifespan when applied onto the wound site, thus preventing infections and promoting inflammatory responses.^112^ To improve the mechanical properties of conductive hydrogels prepared from such polymers, blending with other materials *i.e.* GO or by applying double-network (DN) and triple-network (TN) are some of the strategies presented in the literature.^112,113^ The DN strategy involves the use of interpenetrating polymer networks composed of the brittle conductive polymer, and a second ductile polymer which is cross-linked using nonionic polymer chains.^114^ The TN strategy may be considered as an extension of the DN strategy since, in addition to the conductive polymer, the neutral and concentrated second network is introduced to promote the swelling of the conductive polymer, and a ductile stretchable third network.^115^ In addition to favorable mechanical properties, it is crucial for the conductive hydrogel to possess good swelling properties, since favorable swelling ratios are beneficial to maintaining a moist wound environment and absorbing tissue exudates, which is important for wound repair.^7^ This swelling ratio of the conductive hydrogel is dependent on controlling its pore size and pore structure,^116^ which is dependent on the crosslinking density and the strength of electrostatic interaction between components in the conductive hydrogel.^26^ Furthermore, favorable pore size and pore structure will enhance the mass transfer of cells, cytokines, gas, *etc.*, that are necessary for wound healing.^26^

### 4.3 Antibacterial property

Since bacterial infections can lead to inflammatory reactions at the wound site and thus inhibit the wound healing process, it is necessary for the conductive hydrogel to possess a good antibacterial property.^7^ It is, therefore, possible to enhance the antibacterial property of the conductive hydrogel by introducing antibiotics, cationic polymers, inorganic metals (*i.e.* Ag^+^, Zn^2+^ and Cu^2+^) and metal oxides, to its structure.^39,117^ For instance, in the study by ref. 117, a new conductive hydrogel was prepared *via* the introduction of a medical nano-silver in a composite for the treatment of wound infection. The hydrogel was made based on Ag NPs/CPH material and was shown to have good mechanical properties, healing properties and antibacterial properties when applied to mice models. In another study, Xin Zhao *et al.*,^7^ prepared conductive hydrogel wound dressings using quaternized chitosan-*g*-polyaniline. The quaternized chitosan was shown to have *in situ* antibacterial and excellent inherent antibacterial activity even in non-acidic environments, and promoted cutaneous wound healing. The mechanisms of introducing/enhancing the antibacterial properties of the conductive hydrogel *via* the endogenous antibacterial mechanism, exogenous antibacterial mechanism, and the AgNPs' antibacterial mechanism.^118,119^ The antibacterial properties of conductive hydrogels can also be enhanced by introducing antibacterial agents with photo-responsive antibacterial properties (*e.g.* GO,^25^ CNT,^28^ rGO^29^*etc.*) into the hydrogels. For instance, GO-based conductive hydrogels were shown to aid the disinfection of drug-resistant bacteria (Methicillin-resistant *Staphylococcus aureus*) and thus promote the treatment of infectious wounds.^120^ Similarly, introducing rGO to the hydrogel can endow the conductive hydrogel with photo thermal antibacterial property because of the photothermal effect of rGO.^29^

### 4.4 Hemostatic property

Hemostasis constitutes the initial stage of the wound healing process, with haemorrhage control considered crucial to reducing blood loss in trauma emergency conditions.^121^ Studies have shown that conductive hydrogel wound dressings can be endowed the hemostatic property by relying on physical sealing and absorption of wound extracts to enhance enriched coagulation factors.^122^ The hemostasis effect of conductive hydrogels may be further enhanced by the introduction of cationic polymers, anion, metal, silicon-based materials and polyphenols, to its structure.^39^ For instance,^123^ demonstrated that gelatin-based conductive hydrogels had a favorable hemostatic property. In the study, functionalization of the gelatin using dopamine was undertaken to form a dopamine-grafted gelatin (GelDA), with a mixture 1,4-phenylenebisboronic acid and GO, introduced, such that the conductive GelDA/GO hydrogel was produced *via* the horseradish peroxidase catalytic system. The conductive GelDA/GO hydrogel had excellent tissue adhesion and hemostatic properties when applied to the rat hepatic haemorrhage model.^123^

### 4.5 Self-healing capacity

**Fig. 4:**
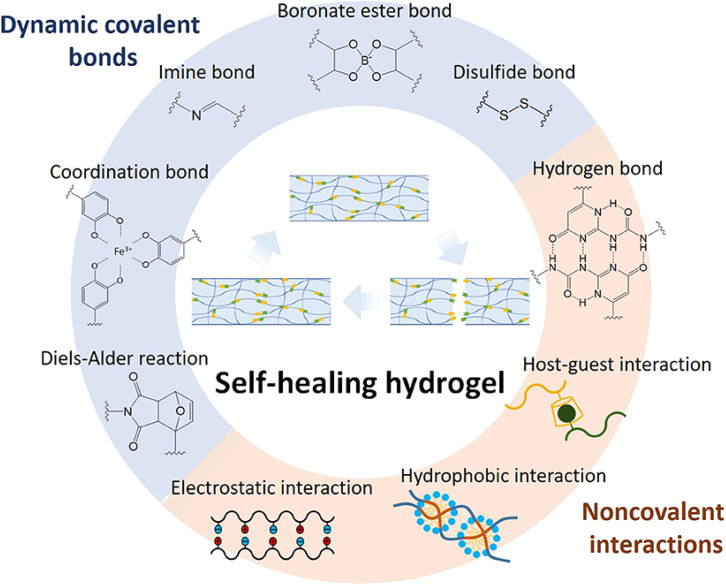
Summary of self-healing mechanisms employed in hydrogels.^126^ This figure has been adapted/reproduced from ref. 126 with permission from *Frontiers in Chemistry*, copyright 2023.

When the wound is exposed to external pressure or regular movement, there is a risk of the breakdown of the hydrogel-based wound dressing. The damage to the wound dressing can lead to bacteria invasion and secondary infections. It is, therefore, important that the conductive hydrogel employed in wound dressing possesses inherent self-healing abilities. This is because, a self-healing conductive hydrogel will have the capability to regenerate *via* dynamic interactions that may be covalent cross-linkages (Schiff-base bonds), covalent, non-covalent, stimuli-responsive (thermal stimulus) and host–guest interactions that ‘recover’ the integrity of the protective layer (Fig. 4).^124–126^

Examples of bonds that can be employed in conductive hydrogels during self-healing are summarized in Table 3.

**Table 3:** Summary of some bonds employed in self-healing mechanisms

Bond	Brief description of the bond	Studies
Dynamic covalent	*Imine formation*: this bond involves the formation of an imine or Schiff base (aliphatic or aromatic) by nucleophilic attack of amine to aldehyde or ketone	127–129
	*Boronate ester complexation*: this is a reversible boronate ester bond that is formed by the complexation of boronic acid and diol	130 and 131
	*Catechol–iron coordination*: this is a reversible coordinate bond that is formed between catechol and iron such that the reversibility of catechol-iron coordination bond can is modulated by controlling the pH conditions	132 and 133
	*Diels–Alder reaction*: it occurs between a conjugated diene and a dienophile, such as an alkene or alkyne, in the absence of a catalyst or coupling reagent	134
	*Disulfide exchange*: this bond is dependent on pH and/or redox potential and occurs *via* disulfide exchange	135 and 136
	*Acylhydrazone bonds*: this bond is formed *via* hydrolysis or exchange reactions and occurs when hydrazine groups react with aldehyde or ketone groups react with hydrazine groups at acidic conditions	137
Non-covalent interactions	*Hydrogen bond*: this is a bond that exists between hydrogen atoms bonded to highly electronegative atoms and another electronegative atom with a lone electron pair	138 and 139
	*Electrostatic interaction*: these are bonds formed from reversible electrostatic interactions existing between charged polymers and ions, polyelectrolytes, polyampholytes and zwitterionic fusions	140 and 141
	*Hydrophobic interaction*: this bond occurs when there is an aggregation of hydro-phobes in an aqueous medium	142 and 143
Multi-mechanism interactions	*Host–guest interaction*: these are bonds that are bonds formed *via* non-covalent interactions, such as van der Waals force occur between different chemical species	144
	*Protein–ligand recognition*: is a bond formed by the interaction between proteins that interact with other molecules or each other and is based on molecular recognition (*i.e.* protein specificity binding of the other molecule)	145

The self-healing conductive hydrogels can prolong the lifespan of wound dressing autonomously self-healing if damaged during the wound healing process.^7^ The development and application of such self-healing conductive hydrogels were demonstrated in the study by ref. 146. In this study, a DN self-healing conductive hydrogel was fabricated using PEG–glycine and PAM–PAA/Fe^3+^ with self-healing properties based on the dual ionic cross-linking within the network. The conductive hydrogel was shown to have favorable mechanical properties highlighted by fracture stress and strain at break values of 0.36 MPa and ∼1350%, respectively. The healed hydrogel was determined to have similar properties to the original hydrogel, with a maximum self-healing efficiency of 97.1% after 12 h.^146^

### 4.6 Stimulus-responsive

**Fig. 5:**
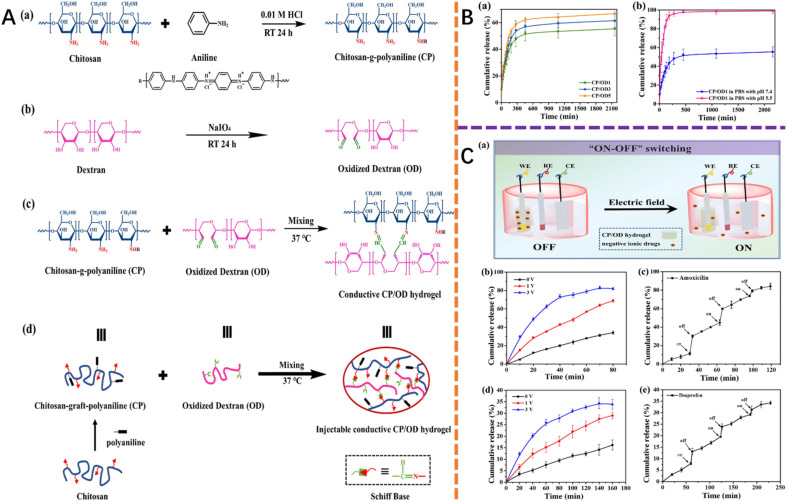
(A) Synthesis of CP copolymer, OD polymer, and CP/OD hydrogel. (B) Drug release of amoxicillin in PBS. (C) The pulse release of drug model from CP/OD conductive hydrogel.^153^ This figure has been adapted/reproduced from ref. 153 with permission from *Acta Biomaterialia*, copyright 2023.

Under external environmental stimuli such as pH, temperature, glucose and light, the stimulation-responsive hydrogels can achieve size or shape changes of different ranges. The temperature-responsive conductive hydrogels are based on polymers with a low critical phase transition temperature (*i.e.* poly(*N*-isopropylacrylamide) with a precipitation temperature of 32 °C).^147,148^ The pH, light and electrical-magnetic field sensitive conductive hydrogels are made from polymers that contain –COOH functional groups, with photothermal effects and conductive-magnetic components, respectively.^149–152^ Such stimulus-responsive conductive hydrogels can be important when employed in the fabrication of wound dressings since they can facilitate control of drug release *via* external stimulus imposition. For instance, in the study,^153^ the release of encapsulated amoxicillin was demonstrated by imposing changes in the pH on the conductive hydrogel prepared by mixing chitosan-*graft*-polyaniline copolymer and oxidized dextran (CP/OD). The study also demonstrated the release of hydrophilic amoxicillin and hydrophobic ibuprofen drugs *via* the imposition of an electrical field (Fig. 5).

### 4.7 Wound monitoring property

In some scenarios, such as diabetic wound ulcers, it may be necessary to facilitate the monitoring of several indicators, such as glucose level, to reduce the risks of medical complications.^154^ Although this monitoring may be achieved *via* the use of commercial glycohemoglobin test kits, the invasiveness of the approach is inconvenient.^154^ The use of conductive hydrogels provides an alternative approach for the real-time and non-invasive monitoring of such cuts.^154^ The use of such conductive hydrogels has the potential to facilitate timely and effective diagnosis and wounds. The use of such conductive hydrogels in wound monitoring has therefore been investigated in the literature.^155^ For instance, in the study by ref. 155, conductive hydrogel prepared by the assembling of polymer networks, based on *in situ* formed poly(tannic acid) (PTA)-doped polypyrrole (PPy) nanofibrils in the poly(acrylamide-acrylated adenine) (P(AM-Aa)) polymer. The study showed that in addition to facilitating visual monitoring of wounds, the conductive hydrogel facilitated enhanced “communication” between cells for accelerated hemostasis, while also enabling the monitoring or detection of the glucose levels on wounds. In another study, novel hydrogel with a conductive property was fabricated from chitosan quaternary ammonium salt (HACC) and sodium alginate (SA), which was employed in wound monitoring.^156^ The hydrogel with a conductivity of 1.14 × 10^−3^ S cm^−1^ was shown to be capable of monitoring shrinkage or joint movement, due to the inherent positive correlation between electrical resistance change and area change. The hydrogel was therefore employed in monitoring wound closure in real-time, using rat models.^156^

### 4.8 Scarless wound healing

**Fig. 6:**
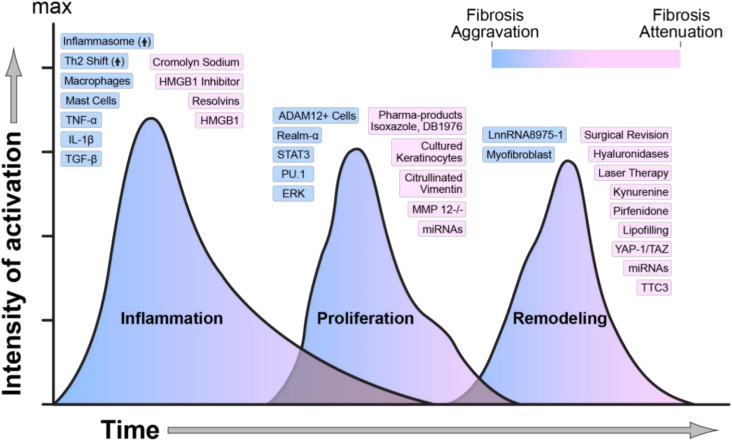
Regulators of wound healing and scarring. Every stage involves numerous molecular, biological, and mechanical factors (blue indicates activation, and pink indicates attenuation of fibrosis).^157^ This figure has been adapted/reproduced from ref. 157 with permission from *International Journal of Molecular Sciences*, copyright 2023.

Existing the difference in wound healing processes between adults and fetal in the extracellular matrix, inflammatory response, cellular mediators, and gene expression profiles. While human embryonic skin wound repair is quick and perfect without scar formation, the adult suffers from slow healing and scars, which is the result of abnormal wound-healing response after skin injury and may bring significant physical and psychological impact for adult patients.^2,157–159^ The process of wound healing and scarring formation involves many molecular, biological, and mechanical factors (Fig. 6).^157^ Although some current clinical strategies have little effect in remedying scarring, such as surgical excision, thermal laser treatment, gene therapy, drug application and so on, it is difficult to completely eliminate scars.^38,157^ As an ideal wound dressing, except for accelerating wound closure and promoting wound healing, it is better that the dressing can reduce scar formation.^7^ Some studies have proved that hydrogel dressings also can avoid scar tissue formation.^27,160–162^

### 4.9 Antioxidant property

Favorable antioxidant properties are important due to their importance in countering inflammation which is responsible for high oxidative stress. The high oxidative stress, arises due to the presence of excessive free radicals that attack cells and destroy proteins in tissues such that high concentrations of reactive oxygen species (ROS) at the wound site leads to a compromise of the fibroblast activity, infections and slow tissue regeneration.^7,31,163,164^ It is, therefore, important that the conductive hydrogels used have antioxidant properties to enable the elimination of free radicals at the wound site. To endow the conductive hydrogel wound dressing with the antioxidant property, materials such as curcumin, dopamine, and honey may be integrated into its structure.^7,39,165^ For instance, in the study by ref. 166, the biocompatible polymer *N*-carboxyethyl chitosan (CEC) was mixed with oxidized hyaluronic acid-*graft*-aniline tetramer (OHA–AT) polymer to produce the conductive hydrogels (OHA–AT/CEC) with anti-oxidant properties. Additionally, *in vivo* experiments showed that the conductive hydrogel accelerated wound healing, leading to excellent collagen disposition and angiogenesis in the skin defect model. Similarly, in the study by ref. 167, a conductive hydrogel was prepared from PANI-modified carboxymethyl chitosan and aldehyde-modified Pluronic F-127 (F127-CHO) using the Schiff base reaction. The conductive hydrogel was determined to have favorable antioxidant activity and Schwann cell proliferation activity when loaded with 7,8-dihydroxyflavone.^167^

### 4.10 3D printability

**Fig. 7:**
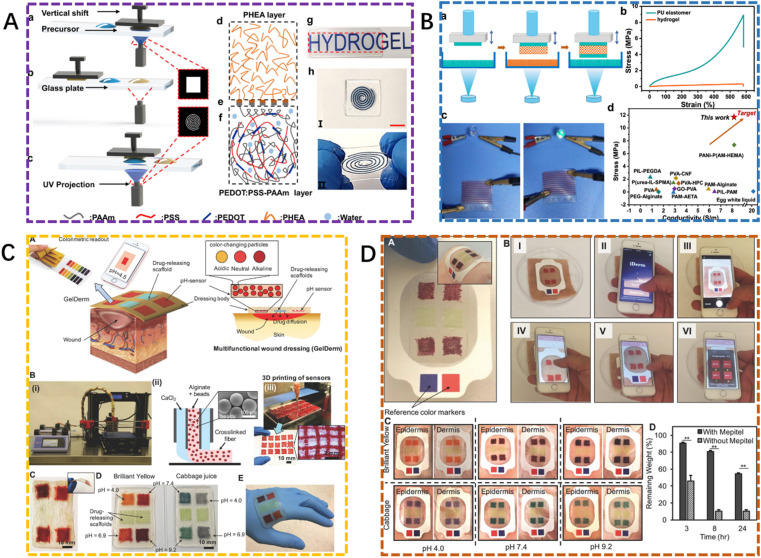
(A) Schematic representation of the additive manufacture of conductive hydrogel–elastomer based on the cross-linking networks in the PHEA layer, PHEA/PEDOT:PSS–PAAm hydrogel interface layer, and PEDOT:PSS–PAAm hydrogel layer.^171^ This figure has been adapted/reproduced from ref. 171 with permission from *Applied Materials*, copyright 2023. (B) Schematic illustration for the dual-material printing process through DLP technology to obtain the packaged hydrogel circuit.^172^ This figure has been adapted/reproduced from ref. 172 with permission from *Applied Materials*, copyright 2023. (C and D) An advanced multifunctional dressing (GelDerm) fabricated by a 3D bioprinter equipped with a co-axial flow microfluidic nozzle for the management of wounds.^173^ These figures have been adapted/reproduced from ref. 173 with permission from *Advanced Healthcare Materials*, copyright 2023.

The conductive hydrogels could be prepared using several methods, including solvent casting, molding, 3D printing, and so on. 3D printing has gained increasing research intention for the fabrication of conductive hydrogels with complex and heterogeneous structures for wound healing dressings, and the different types of 3D printing modalities mainly based on jetting, extrusion, and photopolymerization.^168,169^ Several printable inks with different physiochemical properties, especially 3D printability, have been designed for 3D printing on different devices to fabricate delicate conductive hydrogels. In the 3D printing process, either conductive hydrogel precursors or conductive hydrogels are deposited *via* a nozzle on a substrate of interest.^170^ The ink could be flowed by the applied pressure due to the shear thinning behavior of the ink, and shear forces were introduced to allow the deposition of materials in the desired patterns. Zhu *et al.*,^171^ fabricated 3D functional devices using the digital light processing 3D printing method, the obtained hydrogels possessed high conductivity and superior electrical stability because of the interconnected PEDOT:PSS network. In addition, the strong interfacial bonding between elastomer and hydrogel could be achieved by incomplete photopolymerization, which ensured the stability of the device (Fig. 7A). Similarly, He *et al.*,^172^ prepared a series of conductive hydrogels with high strength (up to 22.9 MPa), ionic conductivity (up to 9.64 S m^−1^), and elasticity (up to 583%) using digital light processing 3D printing technology. Not only the hydrogels with complex structures and a high resolution could be achieved, also the high ionic conductivity for hydrogels could be obtained *via* ion coordination effect and chemical crosslinking (Fig. 7B). In addition, Mirani *et al.*,^173^ reported an advanced multifunctional dressing (GelDerm) that was fabricated by a 3D bioprinter equipped with a co-axial flow microfluidic nozzle. The obtained GelDerm was capable of colorimetric measurement of pH, which could be considered as an indicator of bacterial infection and release of antibiotic agents at the wound site (Fig. 7C and D).

### 4.11 Monitoring property

**Fig. 8:**
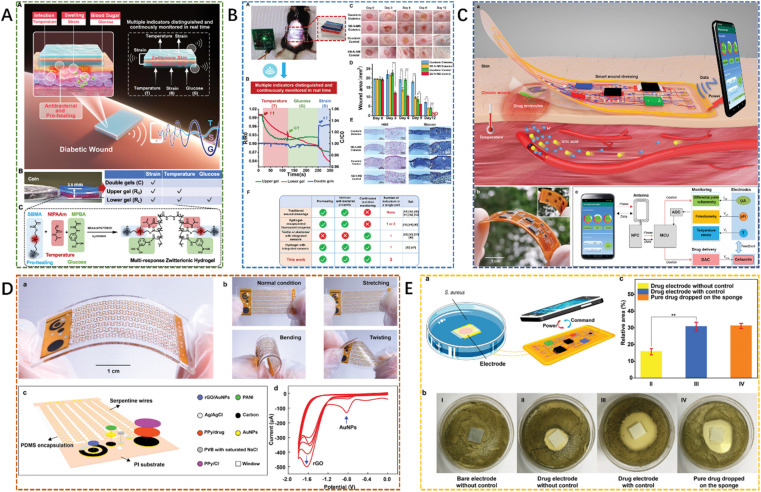
(A) The sandwich-structured sensor based on multi-response zwitterionic skin was designed for multiple sensations and pro-healing of diabetic wounds, and the different signal indicators were measured by double gels, upper gel, and lower gel, respectively. (B) The obtained sandwich-structured sensor could be used for *in vivo* wound healing tests and continuous real-time monitoring on diabetic mouse wounds.^176^ These figures have been adapted/reproduced from ref. 176 with permission from *Advanced Functional Materials*, copyright 2023. (C) The battery-free and wireless wound dressing was fabricated for wound infection monitoring and electrically controlled on-demand drug delivery. The smart dressing was patched on the wound site for simultaneous monitoring of temperature, pH, and uric acid, providing feedback treatment with electrically controlled release of drug molecules. (D) The stretchable electrode array was fabricated. (E) The antibacterial testing system was designed using the Petri dish for culturing *S. aureus*, the drug delivery electrode, the flexible circuit board, and an NFC-enabled smartphone.^177^ These figures have been adapted/reproduced from ref. 177 with permission from *Advanced Functional Materials*, copyright 2023.

Besides the above properties we discussed, the one-time solution strategy could be combined with conductive hydrogel dressings for wound management and monitoring. For instance, Mostafalu *et al.*,^174^ developed a smart and automated flexible wound dressing, temperature and pH sensors were integrated, and the wound status could be real-time monitored to address unmet medical needs. At the same time, the drugs could be on-demand released in a stimuli-responsive drug-releasing system. So, the fabricated wound dressings equipped with a microcontroller could be further programmed to the drug release protocol for individualized treatment (Fig. 8A–C). Pang *et al.*,^175^ designed a smart, flexible electronics-integrated wound dressing with a double-layer structure using polydimethylsiloxane-encapsulated flexible electronics and a UV-responsive antibacterial hydrogel. The obtained integrated wound dressing could provide early infection diagnosis using real-time wound-temperature monitoring, and the on-demand infection treatment could be achieved by the release of antibiotics from the hydrogel (Fig. 8D and E). Similarly, Guo *et al.*,^176^ designed a sandwich-structured skin sensor system based on a zwitterionic thermos-glucose-sensitive hydrogel. The designed wound dressing could be used to pro-heal chronic diabetic wounds with real-time monitoring of infection, swelling, and blood glucose. In another research, Xu *et al.*,^177^ fabricated a wound dressing using conductive hydrogels that integrated with a closed-loop monitoring and treatment system. The integrated near-field communication module through a miniatured circuit and smartphone could realize wireless power harvest and data transmission, on-site signal processing, and drug delivery control.^174,175^

### 4.12 Additional favorable properties of conductive hydrogels

Other properties that would improve the performance of conductive hydrogels in wound dressings include favorable injectability and adhesive properties. Injectability constitutes another important property of conductive hydrogels since favorable injection properties can reduce the secondary injury to the wound in the process of dressing replacement and can completely fill wound parts with different shapes and depths.^178^ A conductive hydrogel with favorable injectability properties also can be used in the encapsulation of drugs. The injectability of a conductive hydrogel can be enhanced when prepared *via* Schiff-base bonds highlighted earlier above. Similarly, a favorable adhesive property of the conductive hydrogel will enhance the wound healing process by sealing the wound and by protecting the wound from the potential risk of infection caused by external microorganisms. Indeed, a non-adherent dressing applied in a wound would require frequent changes of the dressing, which can lead to discomfort for the patient.^30,179^

## 5 Future perspectives

The current review has discussed the functionality of conductive hydrogel dressings as media that can enhance cell signaling for the promotion of cell adhesion, cell proliferation and cell migration for the promotion of wound healing. The use of conductive hydrogels as wound dressings could therefore constitute a healing strategy that can accelerate wound closure and promote skin tissue regeneration. Due to the importance of conductive hydrogels, it is anticipated that future work seeks to enhance and further develop the technology. For instance, studies to improve the understanding of the mechanism of wound healing induced by conductive hydrogel dressing as a basis for developing multifunctional conductive hydrogels, must be promoted. Such multifunctional conductive hydrogels may be endowed with the capacity to facilitate controlled delivery of antibiotics, antibacterial drugs, cells, cytokines, *etc.* to facilitate real-time control and monitoring of wound environment. Improved regulation of the wound environment *via* the control of pH, temperature, glucose, *etc.*, will also facilitate better management of the infected wound and provide objective information about the status of a wound for the patients and doctors. Furthermore, it is anticipated that further work will be undertaken to counter possible reductions in the electrical conductivity of conductive hydrogels overtime, due to the reduction or loss of dopants, thus limiting wound healing progress.^180^ It may be possible to counter these challenges in future *via* the development of more efficient copolymerization, doping, and self-assembly strategies. The authors also predict that future studies will investigate the functionality of employing conductive materials in 3D printing such that personalized printing wound dressing becomes possible. In such a scenario, a model software will be employed in printing the wound dressings which are unique to various patients' wound shapes, thus reducing the potential microbial infection due to imperfect wound protection.^181^ The current study recognizes that current conductive hydrogels have mainly fixed morphology and thus can be only be applied to the surface of wounds, while leaving an internal “cavity” beneath the surface of the wound if it is deep and irregular. The presence of this cavity will limit signal transmission, with the healing effect of the conductive hydrogel, hindered. To resolve the challenge, the use of the so-called adaptive hydrogel will constitute a viable strategy.^182^ Such adaptive conductive hydrogels will possess the ability to spontaneously adjust to environmental factors, such as demonstrating a change in shape to adapt to conditions of irregular or deep, complex wounds. We, therefore, predict that future work will explore the development of these adaptive, conductive hydrogels for enhanced signal transmission in deep, irregular and/or complex wound tissues for effective current flow throughout the wound area for improved therapeutic outcome.^182^ It is anticipated that although most conductive hydrogels are currently in the developmental stage, their advantages will promote their future commercialization for dermal tissue engineering applications. We, however, speculate that of the major conductive hydrogels discussed in this paper, the conductive hydrogels based on PEDOT (*i.e.* PEDOT:PSS) have significant potential for future clinical tests due to their low production cost, excellent mechanical and conductive properties.^183^ Indeed preliminary tests on mice wound conducted *in vivo* support this assertion for the use of PEDOT:PSS as a basis for the development of smart wound dressings for future clinical tests.^184^ More work is, however, necessary to counter the risk of PEDOT chains degradation leading to PSS release, in the long-term.^183^

## 6 Conclusion

Recent works have found that wound dressing hydrogel with conductive properties enables good wound management. This is because such wound dressings can effectively conduct and distribute electrical signals in the wound site to promote the wound-healing process. The review also highlighted the potential of such conductive hydrogels to assist in the regulation of the tissue cellular activities *via* promoting cell migration, proliferation, adhesion, and differentiation when employed as a wound dressing. It was shown that conductive materials could direct/align cell migration from the edge to the wound center, and thus accelerate wound healing and regeneration. The potential to enhance the favorable properties of conductive hydrogels, such as biocompatibility, biodegradation, adhesion, hemostasis, antibiosis and antioxidation properties *via* the introduction of materials such as gelatin, was also discussed. The present review also highlighted the potential of conductive hydrogels to decline over time due to dopant loss which may hinder their positive effects on wound healing, with the need for future studies to address this issue highlighted. Given the importance and potential of conductive hydrogel in the biomedical industry, as well as the global commitment to research in the field, it is expected that the existing limitations of such conductive hydrogels will be addressed for enhanced functionality of conductive hydrogels.

## Author contributions

Lei Nie: conceptualization, software, formal analysis, writing – original draft preparation, writing – reviewing and editing, supervision. Qianqian Wei: writing – original draft preparation. Jingyu Li: writing – original draft preparation. Yaling Deng: methodology, data curation, writing – original draft preparation. Xiaorui He: writing – original draft preparation. Xinyue Gao: writing – original draft preparation. Xiao Ma: writing – original draft preparation. Shuang Liu: writing – original draft preparation. Yanfang Sun: writing – reviewing and editing. Guohua Jiang: writing – reviewing and editing. Okoro Oseweuba Valentine: methodology, writing – original draft preparation, writing – reviewing and editing. Amin Shavandi: writing – original draft preparation, writing – reviewing and editing.

## Conflicts of interest

The authors declare no conflict of interest.

## Supplementary Material
